# Observations of nursing staff compliance to a checklist for person‐centred handovers – a quality improvement project

**DOI:** 10.1111/scs.12686

**Published:** 2019-04-08

**Authors:** Lena Sharp, Carina Dahlén, Mia Bergenmar

**Affiliations:** ^1^ Department of Learning, Informatics, Management and Ethics Karolinska Institutet Stockholm Sweden; ^2^ Regional Cancer Centre Stockholm‐Gotland Sweden; ^3^ Department of Oncology Karolinska University Hospital Stockholm Sweden; ^4^ Department of Oncology‐Pathology Karolinska Institutet Stockholm Sweden; ^5^ Center for Digestive Diseases Karolinska University Hospital Stockholm Sweden

**Keywords:** nursing handover, person‐centred care, clinical information exchange, observations

## Abstract

Nursing shift‐to‐shift handovers are important as they impact the care quality indicators such as safety, patient satisfaction and continuity. However, nurses’ handovers have also been criticised and described as unstructured and ineffective. To improve the handovers and involve patients and their loved ones in the process, a person‐centred handover (PCH) model performed at bedside has been developed and tested at Karolinska University Hospital, Sweden. This study reports on the nursing staffs’ compliance to a checklist used for the newly introduced PCH model. A total of 43 PCH sessions were observed at two acute care wards, using a structured observation protocol. None of the observed handover sessions included all the 13 PCH checklist subcomponents. The checklist was used in 18 (44%) of the observed handover sessions. A statistically significant higher number of subcomponents were observed when the nurses used the PCH checklist (6.4 vs. 4.5 subcomponents, p < 0.05). The mean time spent on each PCH was 6 minutes. In 56% of the sessions, the patients were observed to actively participate in the handover. Overall, the nursing staffs’ compliance to the PCH checklist needs to be improved. The observations suggest that training on communication‐oriented tasks would be beneficial to establish a person‐centred handover process.

## Introduction

Clinical information exchange is an essential part of health care, enhancing quality, continuity, safety, team work and patient‐centredness 1, 2, 3. Clinical staff handovers have been described as ‘the transfer of professional responsibility, and accountability for some aspects of care for a patient, or a group of patients, to another person or professional group on a temporary or permanent basis’ 4.

Nursing shift‐to‐shift handovers, or handoffs, have been recognised as complex and often overlooked parts of the clinical information exchange, which could impact safety, patient satisfaction, continuity and other clinical outcomes 1, 5. However, nursing handovers have also been heavily criticised for being unstructured, ineffective, time‐consuming and not person‐centred 6, 7, 8. The most commonly used nursing handover models 1 are written or verbal handovers, both are often performed at the nurses’ station without direct involvement from patients and/or their loved ones. Bedside handover is a form of verbal handover performed at bedside with patients, their loved ones (if present) and the nursing staff 9.

Two systematic reviews evaluated different handover models 10, 11. The authors of both reviews conclude that no specific model of nursing handover could be recommended over the others. However, it was recommended that nursing handovers should include face‐to‐face information exchange, patient involvement, structured documentation and sufficient information technology (IT) to support the procedure 10.

The traditional view of the patient as a passive recipient of care has been challenged by research findings and also by recent legislation in many countries 12, 13, 14, 15. Despite this, recent Swedish reports indicate insufficient patient participation 16, 17. The terms patient involvement and patient participation are often used interchangeably in the literature. However, Cahill 12 presents in her concept analysis, a 3‐level hierarchical model, with *involvement* being the first level, characterised as a one‐way process where the patients view is often ignored. In the same model, patient *participation* (level 2) is more of a two‐way process where both patients and healthcare providers contribute to the process. *Partnership* represents the highest and, according to Cahill desirable level (level 3), characterised by a joint venture based on a written or verbal contract. Active patient participation is stated to only occur through reciprocal relationships and shared decision‐making between patients and healthcare providers 18. Flink et al. 19 also describe three different levels of patient participation. In their model, level 1 is characterised by a passive patient role while staff having the leading role in the relationship. The second and desirable level (according to Flink et al.) in this model is described as a mutual relationship where the patient and staff are sharing the responsibility and exchanging clinical information that benefits both parts. Both Flink et al. 19 and Cahill 13 point out the benefits of active patient participation in clinical handovers. Handover sessions can be an opportunity for patients to gain information and discuss any health‐related matters with staff. For the staff it could be a daily opportunity to gain first‐hand patient information, including preferences, and hereby improving patient‐centredness.

Person‐centred care (PCC) aims to ensure that patients and healthcare providers are equal partners in care; thus, partnership is considered a key component. However, Määttä et al. 20 discuss the term partnership and possible inherent difficulties due to lack of clarity of the term itself when it comes to implementation of PCC. According to Ekman et al. 21 partnership in PCC is based on mutual agreement between the involved parties and a shift of power in favour of the patient. This can be achieved by including patients and their loved ones in care decisions, with them being experts in their own lives, working alongside healthcare professionals to ensure the best possible outcome. It is the responsibility of healthcare professionals to encourage patients to take on an active role, at the same time taking into consideration the individual capabilities, rights and personal preferences 21. Interventions promoting PCC have shown positive effects on patient satisfaction 22, 23, safety 24 and patient participation 17, 25.

A shift‐to‐shift handover between nursing staff *together* with the patient, person‐centred handover (PCH) was developed, implemented and evaluated at the Department of Oncology, Karolinska University Hospital 26. The PCH model consists of five components (A. preparation, B. introduction, C. information exchange, D. patient involvement and E. safety check) based on the standard operating procedure for implementing bedside handovers 27 and the SBAR (Situation, Background, Assessment, Recommendation) communication protocol 28. Originally developed for inter‐professional communication, SBAR has shown benefits in nurse‐to‐nurse shift handovers, contributing to increased structure, consistency, comprehensiveness and active patient participation 29, 30.

A checklist was developed to support the staff to deliver a structured and standardised information exchange during the PCH sessions. The PCH model was then adapted to a new context. The implementation started with repeated lectures, workshops and role plays (one full day training for all staff at both wards). During these education days, the staff members had the opportunity to discuss, raise concerns and practise the different roles involved in the PCH model. The checklist and information leaflets were modified in collaboration with the staff to suit the new setting. The project progress was also discussed at monthly obligatory staff meetings (at least one of the researchers were also attending) throughout the planning and implementation phase. Observations of the PCH were the next step in the implementation in order to identify any weak points needing further actions/training. The model was then implemented and evaluated at two wards at the Center for Digestive Diseases, at the same hospital in collaboration between the study group and the Department management. Richer et al. 3 describes the importance of adapting handover procedures to local context, for a more successful implementation including compliance to the new model. Time allocated for the preparation before PCH was approximately 30 minutes (for reading of the Electronic Health Records, EHR).

## Aim

The aim of this quality improvement project was to describe and evaluate the nursing staff′s (Registered Nurses, RNs, and nurse assistants, NAs) compliance to the PCH checklist in acute specialist inpatient care.

## Methods

### Setting

The quality improvement project was performed at two wards at a large university hospital. The staff on both wards had the same working schedules, with typical shifts of 8–8.5 hours and with some staff working exclusively night shifts (10‐hour shifts). The two wards were specialised in upper abdominal surgery, hepatology and inflammatory bowel diseases and provided surgical and medical care around the clock for adult patients. Admissions are either acute or planned. Number of beds, median length of stay, work load index, staff ratio, number of nurses (including years of experience) are displayed in Table 1. No changes of the schedules for nursing staff were performed due to the introduction of PCH – the handovers were performed during the overlap between the morning and evening shift. The observations were performed on random and convenient days during a 3‐month period, three to seven months after the introduction of PCH. The days were selected to avoid performing observations on the same weekday repeatedly and also to avoid bank holidays and other days not suitable for observations. For instance, we avoided to perform observations on the same ward too many days in a row as the risk would have been high that many patients had already been observed. Throughout the project, our implementation strategy was close collaboration between the three researchers, managers (the head nurses at each ward and the nurse director) and clinical staff. All these groups were involved in each part of the implementation process.

**Table 1:** Comparison between the two observed wards

	Ward X	Ward Y
Ward description		
Number of beds	18	12
Length of stay, median days	8.6	5
Time since introduction of PCH, months	3	7
Workload index^a^	3.9	3.1
Staff ratio^b^	1.94	1.66
Years of experience among RNs (non‐agency staff)		
<1 year	4	1
1–3 years	2	2
>3 years	5	2

PCH, person‐centred handover; RN, Registered Nurses.

a According to a locally developed programme for registration of work load based on admitted patients’ medical conditions and nursing care needs. Scores ranging from 2 (patient managing their own care and in need of minimal staff attention) to 6 (patient with extensive care needs and complex medical treatment). The figures reflect each wards mean daily total score during the observation period.

b Number of budgeted nursing staff (RNs and nurse assistants) per bed.

### Observation

Data were collected through direct and structured observations. The focus was aimed at the nursing staff's compliance to the checklist during PCHs. Each observation followed the same structure outlined in an observation protocol, developed by the study group. The protocol covered the content of the PCH model. This included 13 items which were divided into five components and subcomponents (A1 preparation; B1‐2 introduction; C1‐3 information exchange; D1‐3 patient involvement; E1‐6 safety check), Table 2. Performed PCH components together with notes made (on time taken for each session, number of persons involved, whether the PCH checklist was used (yes/no), number of and reasons for interruptions) were documented in the observation protocol for each PCH. Agency nursing staff were not included in the observations.

**Table 2:** General observations and observed checklist components of the person‐centred handover (PCH) sessions

	Ward X	Ward Y	Total
	n (%)	n (%)	n (%)
General observations			
Observed sessions	23 (53)	20 (47)	43 (100)
Sessions where patients loved one were present?	1 (4)	3 (15)	4 (9)
Paper version of PCH checklist present during session	0 (0)	18 (90)	18 (42)
Sessions interrupted	3 (13)^b^	4 (20)^b^	7 (16)^b^
	Min‐Max	Min‐Max	Min‐Max
Number of staff participating	2–8	2–5	3–8
	(min‐max)	(min‐max)	(min‐max)
Mean time spent on PCH sessions, minutes	5 (2–7)	7 (4–13)	6 (2–13)
PCH components (Items on the checklist)			
A. Preparation			
Patients were informed about the upcoming handover prior to the PCH session (A1)	2 (9)	2 (10)	4 (9)
B. Introduction (at bedside)			
Staff introducing themselves to patient and present loved ones (B1)^a^	14 (61)	19 (95)	33 (77)
The patient is invited to report any care‐related matters (B2)	22 (96)	18 (90)	40 (93)
C. Information exchange (at bedside)			
Planned care activities for the next 24 hours discussed with patient and present loved ones (C1)	17 (74)	20 (100)	37 (86)
Medical jargon is avoided (C2)	23 (100)	20 (100)	43 (100)
The oncoming nurse summarises and concludes the information exchange and confirms with the patient, loved ones and colleagues (C3)	0 (0)	3 (15)	3 (7)
D. Level of patients’ involvement in the PCH (at bedside)			
Passive patient, staff having the leading role (D1)	6 (26)	1 (5)	7 (16)
Patient and staff sharing the responsibility, information exchange (D2)	10 (44)	15 (75)	25 (58)
Active patient having the leading role (D3)	7 (30)	4 (20)	11 (26)
E. Safety check (at bedside)			
Staff check that the patient has a correct ID wrist band (E1)	11 (48)	19 (95)	30 (70)
Fall risk discussed with the patient (E2)	3 (13)	11 (55)	14 (33)
Check ongoing infusions, when relevant (E3)	0 of 3	5 of 6	5 of 9 (56)
Ordered medications changes are checked and confirm with the patient and colleagues (E4)	4 (17)	7 (35)	11 (26)
Check if the patient or loved ones have any questions/concerns related to medications (E5)	0	3 (15)	3 (7)
The patient and/or loved ones are asked if they have any safety concerns or have noticed anything divergence (E6)	2 (9)	9 (45)	11 (26)

EHR = electronic health record; PCH = person‐centred handover; RNs = Registered Nurses.

a Depending on if the patients were known to the RN from earlier shift or not.

b By late coming or early leaving PCH staff (n = 4), by personal assistant speaking on behalf of but without permission from patient (n = 1) or by staff not involved in the PCH session (n = 2).

The level of patient participation was categorised as ‘passive patient, staff having the leading role’ (D1), ‘patient and staff sharing the responsibility, information exchange’ (D2) or ‘active patient having the leading role (D3)’ according to Flink et al. 19.

All observations were performed by the same specialist nurse, who had previous experience of PCH. She was present, as part of the nursing team, during the whole PCH session without interfering in the actual procedure. This nurse was a permanent staff member and was therefore familiar with routines and organisation. She was, however, employed at another hospital site, which means that she did not know the staff in person or the patients on the wards where she performed the observations. No patient was observed more than once. The observer kept a list of observed patients, only including patients initials, birth year, ward and date of observation. This list was kept in a locked cabinet and was at all time only available to the observer. After the data collection was completed, the observer destroyed the list.

### Ethical considerations

In this quality improvement project, data were collected by observations, the same method that is used for collecting data for other quality indicators, such as nurse‐sensitive outcomes. For example, nurse‐patient interaction related to drug administrations is regularly observed, in accordance with Swedish national care quality guidelines. The focus in both cases is the professionals, rather than the patients. Consequently, no data on the patients’ characteristics were collected in this project. The PCH model was already established before data collection and formed the bases for the evaluation. However, at admission, all patients received a written information describing the PCH including a paragraph on voluntariness and the possibility to opt out. This information also included the patients loved ones, in case they were present during handovers. The nursing staff gave their verbal consent prior to each observation. Despite the absence of an informed consent procedure, all precautions were taken to protect patient integrity during the observations. No formal ethical approval was required since the project was part of an evaluation of a standard care procedure. The project was approved by the Head of the Department.

### Data analysis

The frequencies and proportions of the observed PCH checklist components, level of patient participation and the other items in the observation protocol were summarised and presented descriptively. Unpaired t‐tests were performed testing the differences of the use of the checklists (yes/no) on time taken for PCH at bedside (with or without checklist) and the number of performed components (A‐E) during the PCR (with or without checklist).

## Results

A total of 43 PCH observations were performed during March to May 2016. The differences between the wards regarding the number of beds, length of stay, workload, staff ratio, RNs education level and years of experience are presented in Table 1. None of the 43 PCH sessions included all 13 subcomponents on the PCH checklist. The checklist was used on all but two of the observed sessions on Ward Y, while not at all on Ward X. A statistically significant higher number of subcomponents included in PCH sessions were observed when the checklist was used (6.4, SD 1.7 vs. 4.5 subcomponents, SD 1.3, p < 0.05).

The mean time spent on PCH sessions at bedside was 6 minutes (ranging between 2–13 minutes). When the checklist was used, the mean time was 6.7 minutes, SD 2.1, compared to 4.8, SD 1.3 if the checklist was not used (p < 0.005).

**Figure 1:**
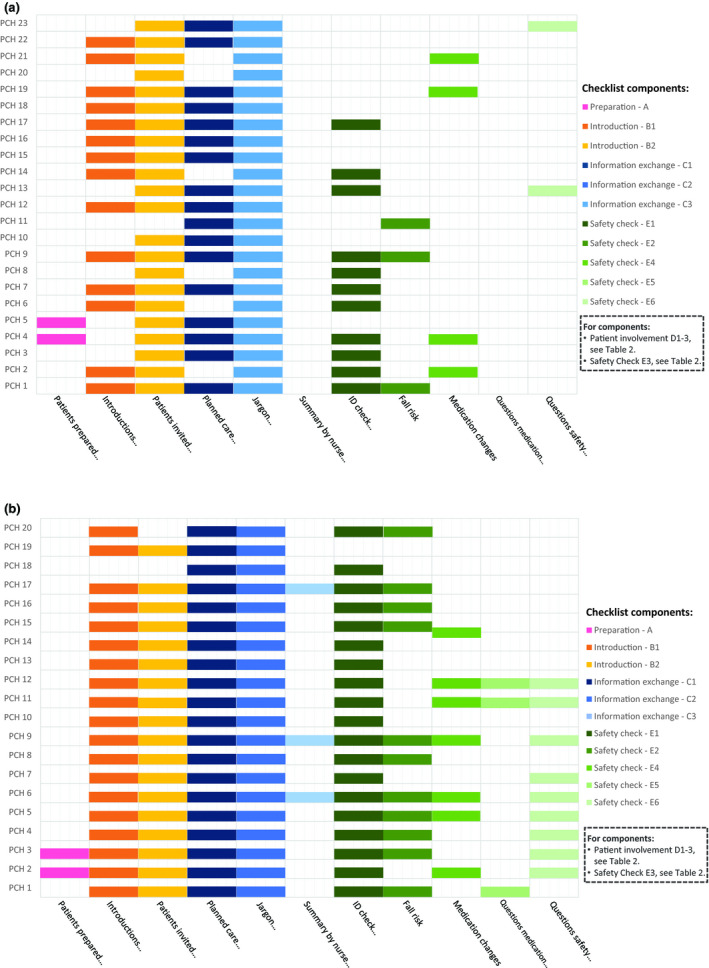
(a) Observed checklist components Ward X. (b) Observed checklist components Ward Y.

The performed components of each PCH session (A, B, C and E with subcomponents) are presented in Fig. 1a (Ward X) and 1b (Ward Y). Results for component D and subcomponent E3 are displayed in Table 2.

The majority of the patients (91%) were not informed and/or reminded of the handover procedure prior to the PCH session (component A). In most cases, the introductions (component B1‐2) were performed according to the checklist. The number of participating staff (RNs, NAs and nursing students) during the handover ranged from 3 to 8. Data on staff and loved ones present, time spent on PCH components B‐E, presence of PCH checklists and numbers of interrupted PCHs per ward are presented in Table 2. Compliance to component C (information exchange) varied, with a higher compliance for ‘planned care activities’ (C1) and ‘medical jargon avoided’ (C2) but lower for ‘the summery by nurse’ (C3).

For the six subcomponents E, safety check, substantial differences in compliance were identified and were higher when the checklist was used (2.3 vs. 1.1). The highest compliance was found for task‐oriented assignments (checking wrist bands for ID, E1, and ongoing infusions, E3). In 9 (21%) of the PCH sessions, the patients had ongoing infusions. Five of these (56%) were not checked against the physician's instructions (ID, content, dose, speed), Table 2. Few of the sessions included communication‐oriented components, for example controlling whether the patient or their loved ones had any questions/concerns related to medications (E5), or if they had noticed any divergence (E6).

For 25 (58%) of the PCH sessions, the level of patient participation was categorised as level 2, for example ‘patient and staff sharing the responsibility’ (Table 2). The level 2 sessions (D2) lasted about one minute longer (mean 6.1 minutes, SD 1.4) than the level 1 (D1 = 5.1 minutes, SD 3.6) and level 3 (D3 = 4.8 minutes, SD 1.0) sessions. We found no association between number of performed PCH components and the level of patient participation.

## Discussion

We found insufficient compliance to the handover checklist in this quality improvement project of 43 bedside nursing handover sessions, as none of the observed PCH sessions included all of the checklist subcomponents. An important finding was that the handover checklist was only used in 18 (44%) of the observed PCH sessions, which is surprising considering the observations were done relatively shortly after the introduction of the new handover model. We also found that more of the subcomponents were included if the checklist was used, but even then, only about half of the subcomponents were observed, leaving room for major improvements. Philpin 31 points out the importance of using a supportive tool during bedside handovers. She found, in her qualitative study, that intensive care nurses regarded an observation chart as useful for the complex information exchange during bedside handovers and describe it as an important tool in organising and structuring the handover process. Tobiano et al. 32 conclude in their qualitative study that a more standardised process would help nurses overcome the barriers they perceived with bedside handovers. A bedside handover checklist could contribute to this structure. Sexton et al. 6 suggest that the degree of structure on a wards handover routines also could impact both quality of care in addition to job satisfaction. It has also been reported that patients regarded the nurses’ use of documents during bedside handover as reassuring 13. Not using the PCH checklist may increase the risk of only bringing the traditional version of the handover, from the nurses’ station to the bedside, rather than changing to a person‐centred handover.

Only 9% of the patients were informed beforehand that the PCH session would take place (component A) and thus had the possibility of knowing what was expected from them. It is reasonable to assume that this had negative consequences on active patient participation. Other studies on clinical handovers highlight the importance of patients and their present loved ones clearly understanding the purpose and that active participation is expected 8, 19, 32. Cahill 13 has studied patients’ perceptions of bedside handovers and state that they wished for a better understanding concerning the role they were expected to take. This was also supported by Timonen and Sihvonen 33, who found that one‐third of the patients thought that the purpose of the bedside handover was meant for nurse‐to‐nurse communication and did not feel that they were expected to be actively involved.

The second component in the PCH session was the ‘Introduction’ (B1‐2). While most sessions (93%) included the subcomponent B2 (The patient being invited to report any care‐related matters), far fewer sessions (77%) included B1 (Staff introducing themselves to patient and their present loved ones). The proportion of the observed PCH sessions not including B1 (23%) was surprisingly high considering this is a task done both quickly and easily and should be an obvious start to an effective and respectful handover process. While all observed PCH session included C2 (Medical jargon is avoided), 86% included subcomponent C1 (Planned care activities for the next 24 hours discussed with patient and present loved ones) and only 7% included C3 (The oncoming nurse summarises and concludes the information exchange and confirms with the patient, loved ones and colleagues). This is one of the subcomponents that require advanced communication skills and familiarity with the procedure. The fact that this summary most often was missing may inhibit an effective information exchange and could create a barrier for active patient participation. Another barrier could be that the equal partnership, described as a key component in PCC (and the highest and most desirable level of patient participation) according to Cahill 12, was not established. Määttä et al. 20 questions if equal partnership between patients and healthcare providers is desirable or even possible. In comparison with other studies 34, 35, the proportion of PCH sessions in which patient participation reached shared responsibility and information exchange (D2) seem to be higher (58%). One explanation could be that the purpose of the new handover model was to increase active patient participation with the PCR‐checklist inviting the patient to report any care‐related matters (B2). The relatively high proportion (42%) of handovers in which shared responsibility was not observed might also negatively have impacted on patient participation.

The final component of the PCH, safety (E1‐6), was also the most poorly performed. The two task‐oriented safety components (ID‐check and check of ongoing infusions, E1 and E3) were more often performed, compared to the communication‐oriented safety subcomponents (E2, E4‐6). Chen et al. 36 also found in their qualitative study that nurses in acute care settings prioritised more practical tasks. The same authors conclude that this was a barrier for effective communication and a missed opportunity to improve both safety and patient involvement on a daily basis. The short time span between the introduction of PCH and the observations may possibly explain the prioritisation of task‐oriented subcomponents. This could be a reason for the consistent lower performance rate of all subcomponents requiring communication. Nurses’ communication skills are a key component that can be developed and improved with education and experience. Both Drach‐Zahavy and Shilman 35 and Tobiano et al. 32 concludes that nurses’ communication skills need to be improved to facilitate patient participation during bedside handovers. Drach‐Zahavy and Shilman 35 also found that the information exchange improved, if the patients loved ones were present. In a study comparing patients’ and nurse's preferences, having a loved one present during the handover was reported as important to patients, but not considered important among the nurses 37. In our study, only 9% of the patients had a loved one present. A possible way to improve communication would therefore be to encouraged partners and/or other loved ones to take an active role here.

The mean time spent on each PCH session in our study was 6 minutes. Timonen and Sihvonen 33 observed a mean time of 3 minutes in their study from surgical hospital wards, while Philpin 31 observed 15 min for the bedside handover sessions in her study from intensive care wards. Bedside handovers have been described as both time‐consuming and time‐effective 7, 24. The previous nursing handovers in our study (at the nurse station) typically took 1 hour. With a maximum of six patients per nurse being routine on these wards (including the 30 minutes allowed for reading in preparation), the total time spent for PCH would be approximately be the same.

With improved communication skills, the nurses would hopefully have the opportunity to spend a larger proportion of the PCH session at bedside, especially if available IT support is used (e.g. a laptop available in the patient room during PCH for access to the EHR including digital medication instructions) as recommended by Smeulers et al. 10. Our previous research 26 has shown that even if the PCH sessions are relatively short (and only take place once a day), patients notice a positive difference (significantly higher scores on information exchange) compared with traditional nursing handovers.

### Strengths and weaknesses

Many nursing interventions are implemented without a robust evaluation. This includes the compliance to the various components of an intervention, making evaluations difficult 5, 28, 38. Tobiano et al. conclude in their systematic review 39 that evaluations of quality improvement projects on bedside report are important, to better understand the processes involved. This evaluation has helped us better understand the processes involved in PCH, their strengths and weaknesses and has given us important knowledge on how to improve the quality of the nursing handovers.

The plan to evaluate this intervention through observations with focus on staff compliance was decided on by the two wards nursing teams and managers before PCH was introduced. Scheidenhelm and Reitz 40 also found low nursing staff compliance to the bedside handover process. After their SWOT (strengths, weaknesses, opportunities and threats) analysis and a changed management strategy, bedside handovers were reintroduced. The evaluation (random observations) showed clear improvements with considerably higher compliance. A similar process was planned for the wards in our study.

Observational studies have been described as an option to capture verbal communication in clinical settings, for example at bedside handovers 41. In this project, a relatively small number of nursing handover sessions were observed. However, previous research 42 indicates that approximately 20 episodes are needed to capture the variation in key components of handover sessions. The project was limited to checking the nurses’ compliance to the PCH checklist, excluding information on performance such as body language, tone of voice and also experiences. Data on both the patients’ and the nurses’ experiences 43 from PCH have been collected from the Department of Oncology (where PCH was originally developed). Both these perspectives will need to be addressed for a more comprehensive understanding of the effect of the PCH model. As this was an observational study, we did not collect data on work place culture/climate. Research has shown that the culture at a work place can influence the success of clinical quality improvement projects 44.

Observations in clinical settings can be perceived as more intrusive compared to, for instance, questionnaires. In addition, there is a risk that the observed persons, in this project the nurses, alter their behaviours just because they know they are observed. However, observations can capture actions and communication skills that otherwise would be difficult to catch using other types of data collections that are more sensitive to social desirability (self‐reports). This may be the reason why observations are frequently used for measuring quality care indicators. In this project, the observer was familiar with the setting, the PCH procedure and the staff. We believe that these circumstances contributed to that the observations were perceived as less threatening than if they had been performed by an external observer.

To ensure as high reliability as possible, we used a structured observation protocol covering all the PCH components. The observer was a specialist nurse with previous experience using the PCH model. She was trained to perform the observations by two experienced nursing researchers. All the nurses at both wards were observed at least once, and the observations were performed at random days to allow for differences in staff‐mix and workload.

## Conclusion

This evaluation (made shortly after the introduction of the PCH) shows that the nursing staffs’ compliance to the handover checklist needs improvements and highlights the need for greater focus on communication‐oriented tasks during handovers. More attention is needed on person‐centred information exchange between the patients and nursing staff. The use of the PCH checklist contributes to a more standardised and comprehensive handover procedure in which nurses encourage both patients and their loved ones to take an active role.

## Conflict of interest

The authors declare that there is no conflict of interest.

## Author contributions

Lena Sharp (LS), Carina Dahlén (CD) and Mia Bergenmar (MB) contributed to the study conception and design. CD contributed to the data collection. LS, CD and MB contributed to the data analyses and interpretation and the writing and editing of the article.

## Ethical approval

This quality improvement project was approved by the Head of the Department. Quality improvement project does not require ethical approval.

## Funding

The authors received funding (research time) from the following sources; Regional Cancer Center, Stockholm‐Gotland, Center for Digestive Diseases at Karolinska University Hospital and Swedish Cancer Society.
